# Four differentially expressed genes can predict prognosis and microenvironment immune infiltration in lung cancer: a study based on data from the GEO

**DOI:** 10.1186/s12885-022-09296-8

**Published:** 2022-02-21

**Authors:** Shaodi Wen, Weiwei Peng, Yuzhong Chen, Xiaoyue Du, Jingwei Xia, Bo Shen, Guoren Zhou

**Affiliations:** The Affiliated Cancer Hospital of Nanjing Medical University, Jiangsu Cancer Hospital, Jiangsu Institute of Cancer Research, Nanjing, China

**Keywords:** Lung cancer, Immune infiltrate, Prognosis, Immune-related genes, Gene Expression Omnibus

## Abstract

**Background:**

Lung cancer is among the major diseases threatening human health. Although the immune response plays an important role in tumor development, its exact mechanisms are unclear.

**Materials and methods:**

Here, we used CIBERSORT and ESTIMATE algorithms to determine the proportion of tumor-infiltrating immune cells (TICs) as well as the number of immune and mesenchymal components from the data of 474 lung cancer patients from the Gene Expression Omnibus database. And we used data from The Cancer Genome Atlas database (TCGA) for validation.

**Results:**

We observed that immune, stromal, and assessment scores were only somewhat related to survival with no statistically significant differences. Further investigations revealed these scores to be associated with different pathology types. GO and KEGG analyses of differentially expressed genes revealed that they were strongly associated with immunity in lung cancer. In order to determine whether the signaling pathways identified by GO and KEGG signaling pathway enrichment analyses were up- or down-regulated, we performed a gene set enrichment analysis using the entire matrix of differentially expressed genes. We found that signaling pathways involved in hallmark allograft rejection, hallmark apical junction, hallmark interferon gamma response, the hallmark P53 pathway, and the hallmark TNF-α signaling via NF-ĸB were up-regulated in the high-ESTIMATE-score group. CIBERSORT analysis for the proportion of TICs revealed that different immune cells were positively correlated with the ESTIMATE score. Cox regression analysis of the differentially expressed genes revealed that *CPA3*, *C15orf48*, *FCGR1B*, and *GNG4* were associated with patient prognosis. A prognostic model was constructed wherein patients with high-risk scores had a worse prognosis (*p* < 0.001 using the log-rank test). The Area Under Curve (AUC)value for the risk model in predicting the survival was 0.666. The validation set C index was 0.631 (95% CI: 0.580–0.652). The AUC for the risk formula in the validation set was 0.560 that confirmed predictivity of the signature.

**Conclusion:**

We found that immune-related gene expression models could predict patient prognosis. Moreover, high- and low-ESTIMATE-score groups had different types of immune cell infiltration.

**Supplementary Information:**

The online version contains supplementary material available at 10.1186/s12885-022-09296-8.

## Introduction

Cancer is a leading cause of death and a major hurdle in increasing life expectancy in every country of the world [1]. According to estimates from the World Health Organization (WHO) in 2019, cancer is the either the first or the second leading cause of death before the age of 70 years in 112 of 183 countries; it ranks third or fourth in another 23 countries [2]. Global cancer statistics 2020 [3] showed that lung cancer (11.4% of total cancer cases in 2020) was second to only female breast cancer, which is the most commonly diagnosed cancer (11.7% of total cancer cases in 2020). In addition to being the leading cause of cancer death in men in 93 countries, lung cancer was also the leading cause of cancer death in 2020, representing approximately one in 10 (11.4%) cancers diagnosed and one in five (18.0%) deaths recorded. The primary treatments for lung cancer currently include surgery, targeted therapy, radiotherapy, chemotherapy, and immunotherapy. The advent of immune checkpoint inhibitors (ICIs)that target programmed death-1(PD-1)/programmed death-ligand 1 (PD-L1) has broadened the treatment options for lung cancer patients [4]. They are characterized by low toxicity, great efficacy, and long-term benefits when effective [5]. However, ICIs present many therapeutic problems that seem to be related to individual differences and the complex microenvironment of tumors [6].

Tumor microenvironment has been reported to have a significant impact on the immune response [7]. After receiving immunotherapy for lung cancer, a proportion of patients in the clinic did not respond to immunotherapy and others developed resistance, called ‘acquired resistance’ [8], after they had an initial immune response [9–11]. There are various mechanisms of resistance to immunotherapy that are currently being studied. These include the following: (i) activation of oncogenic pathways; (ii) the IFN-γ signaling pathway; (iii) defects in tumor neoantigen expression/presentation or neoantigen depletion; (iv) additional inhibitory checkpoints; and (v) phenotype transformation [12–16]. Although different treatments lead to different clinical outcomes, the immune cell infiltration status of the tumor microenvironment has an impact on not only the prognosis of lung cancer patients, but also the efficacy of immunotherapy.

Therefore, we propose an assessment of the tumor microenvironment immune infiltration status at the gene expression level to analyze the impact of immune and stromal scores on the patients, thereby identifying immune-related genes that can predict and assess the prognosis and the immune infiltration status in different pathological types of lung cancer patients.

## Methods

### Data of lung cancer cohorts

Whole-transcriptome RNA-seq data for 474 lung cancer cases and their corresponding clinical data were downloaded from the Gene Expression Omnibus (GEO). Datasets GSE30219 and GSE50081 (based on the GPL570 platform) were downloaded from the GEO website (https://www.ncbi.nlm.nih.gov/geo) and analyzed. We downloaded 1008 data of lung squamous carcinoma and lung adenocarcinoma from the TCGA database (https://portal.gdc.cancer.gov/), and 1/5 of the total number was selected by random sampling, and the information of 182 NSCLC patients was finally obtained after removing the paracancerous tissues. These cases were used as validation set data for model building.

### Computation of immune score, stromal score, and ESTIMATE score

An algorithm called ESTIMATE [17] was used to estimate stromal and immune cells in malignant tumor tissues based on the gene expression data [18]. The stromal scores, immune scores, and ESTIMATE scores were calculated for each sample. Lung cancer gene expression data and clinical information comprising 39 basaloid (BAS) samples, 23 carcinoids tumors (CARCI) samples, 63 large cell tumor (LCC) samples, 213 lung adenocarcinoma (LUAD) samples, 106 lung squamous cell carcinoma (LUSC) samples, and 17 small cell carcinoma tissue (SCC) samples were also downloaded. Clinical information including age, sex, tumor grade, pathological stage, AJCC TNM stage, and survival outcome was all obtained from the GEO database. For comparisons between two groups, statistical significance for normally distributed variables was estimated using the unpaired Student’s t-test, and non-normally distributed variables were analyzed using the Mann–Whitney U test. Correlation coefficients were computed using Spearman analyses.

### Identification of differentially expressed genes, gene set enrichment analysis, and PPI network construction

All the 474 tumor samples were classified as high- or low-score based on their comparison with the median Immune score, Stromal score, and ESTIMATE score. The ‘limma’ R package was used to identify immune-related differentially expressed genes (DEGs) [19], and all DEGs with an FDR lower than 0.05 and a |log FC| of more than 0.5 were shortlisted. The gene ontology (GO) pathway enrichment analysis and KOBAS-Kyoto Encyclopedia of Genes and Genomes (KEGG) pathway enrichment analysis of DEGs were performed using the ‘clusterProfiler, org.Hs.eg.db, plot, ggplot2’ packages in R [20]. The Protein–protein interaction (PPI) network of the DEGs was constructed using the Bioinformatics & Evolutionary Genomics online database by determining interactions among DEGs.

### Survival analysis and cox regression analysis

Detailed records on the survival time for the 474 cases were used along with the R packages ‘survival’ and ‘survminer’ to perform the survival analysis. We used mean values for grouping. The survival curve was plotted using the Kaplan–Meier method and the log rank test was used to determine statistical significance; *p* < 0.05 was considered statistically significant. Next, we constructed a multivariate Cox proportional risk regression model based on immune-related DEGs. The risk scores were calculated using the following formula: risk score = expression of gene1 × β1gene1 + expression of gene2 × β2gene2 + ... + expression of genen × βngenen. The best risk score was used as a cut-off to divide the patients into high- or low-risk groups. The predictive ability of the risk model was evaluated through a time-dependent receiver operating characteristic (ROC) analysis. Then, we used 182 NSCLC patients from TCGA as validation set, calculated risk scores based on the coefficients after modeling, and assessed the predictive power of the validation set using C-index and ROC curves.

### Profile of tumor-infiltrating immune cells (TICs)

CIBERSORT is a deconvolution algorithm to estimate cell composition of tissues based on their gene expression profiles [21]. We used CIBERSORT (http://cibersort.stanford.edu/) to examine the relative proportions of 22 types of infiltrating immune cells in the matrix. The algorithm used a default signature matrix with 100 permutations. Only data with p-values lower than 0.05 from the CIBERSORT analysis were selected for subsequent analyses. The distributions of immune cell subsets in each sample are presented as box plots.

## Results

### The flowchart of this study

The analysis process used in this study is shown in Supplement Fig. 1. In order to identify differential gene expression in the tumor microenvironment and detect the relationship between DEGs and prognosis, we downloaded two RNA-seq datasets for lung cancer patients with prognostic data. In total, 474 cases with complete prognostic information and clinicopathological data were collected. After categorizing the patients' microenvironment immune infiltration status based on Immune score, Stromal score, and ESTIMATE score, we explored the relationship between these different scores and basic clinical features, prognosis, pathogenesis, as well as cellular composition of the microenvironment. The basic characteristics of the patients were shown in Supplement Table 1.

### Immune score, stromal score, and ESTIMATE score were associated with clinical features and prognostics of lung cancer patients

**Fig. 1:**
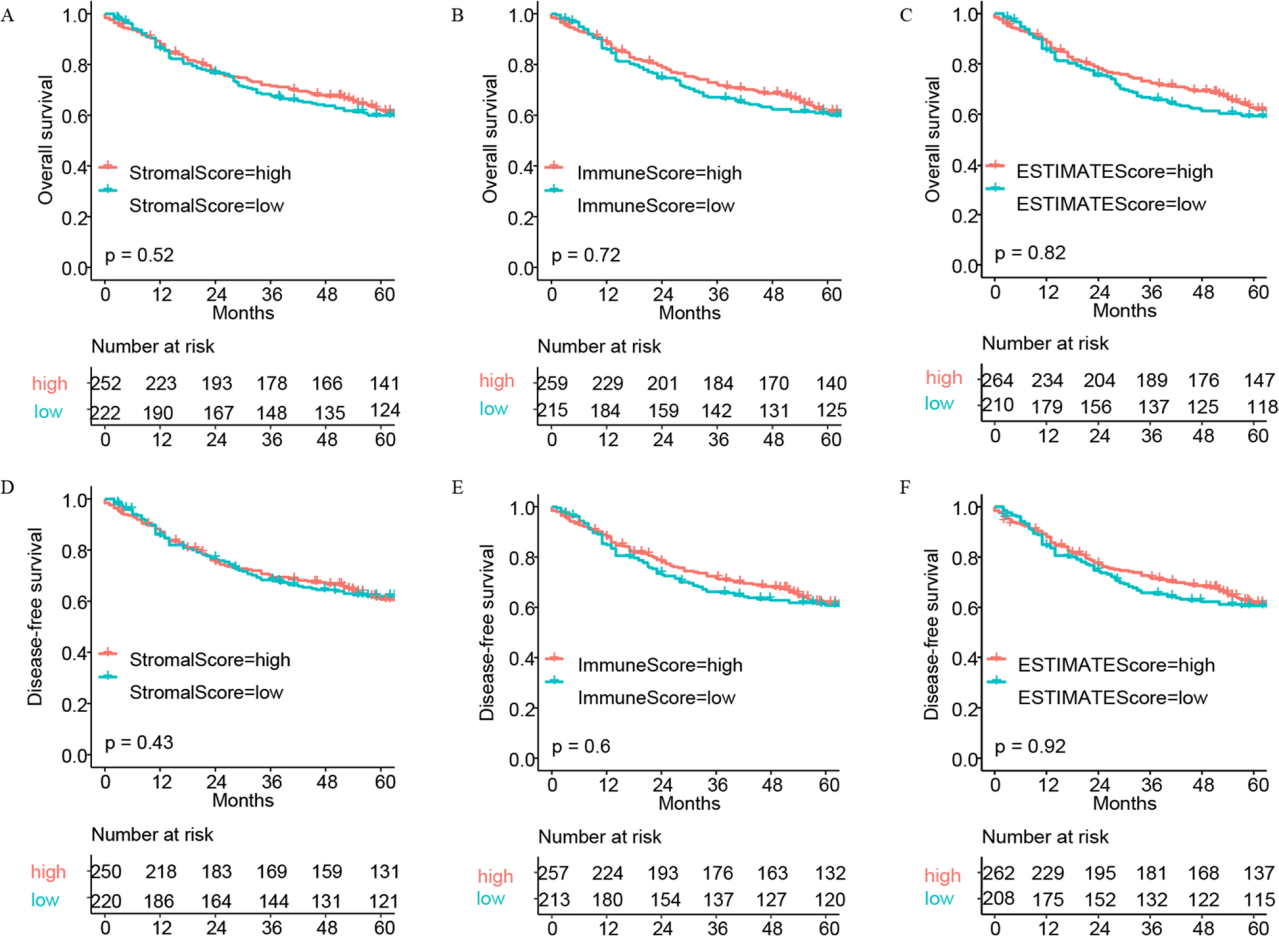
Correlation of the different scores with the survival of lung cancer patients. **A** Kaplan–Meier survival analysis for lung cancer patients classified into high- or low-Stromal-score groups based on comparison with the median (*p* = 0.52 by log-rank test). **B** Kaplan–Meier survival analysis for lung cancer patients classified into high- or low-Immune-score groups based on comparison with the median (*p* = 0.72 by log-rank test). **C** Kaplan–Meier survival analysis for lung cancer patients classified into high- or low-ESTIMATE-score groups based on comparison with the median (*p* = 0.82 by log-rank test). **D** Disease free survival analysis for lung cancer patients classified into high- or low-Stromal-score groups based on comparison with the median (*p* = 0.43 by log-rank test). **E** Disease free survival analysis for lung cancer patients classified into high- or low-Immune-score groups based on comparison with the median (*p* = 0.60 by log-rank test). **F** Disease free survival analysis for lung cancer patients classified into high- or low-ESTIMATE-score groups based on comparison with the median (*p* = 0.82 by log-rank test)

**Fig. 2:**
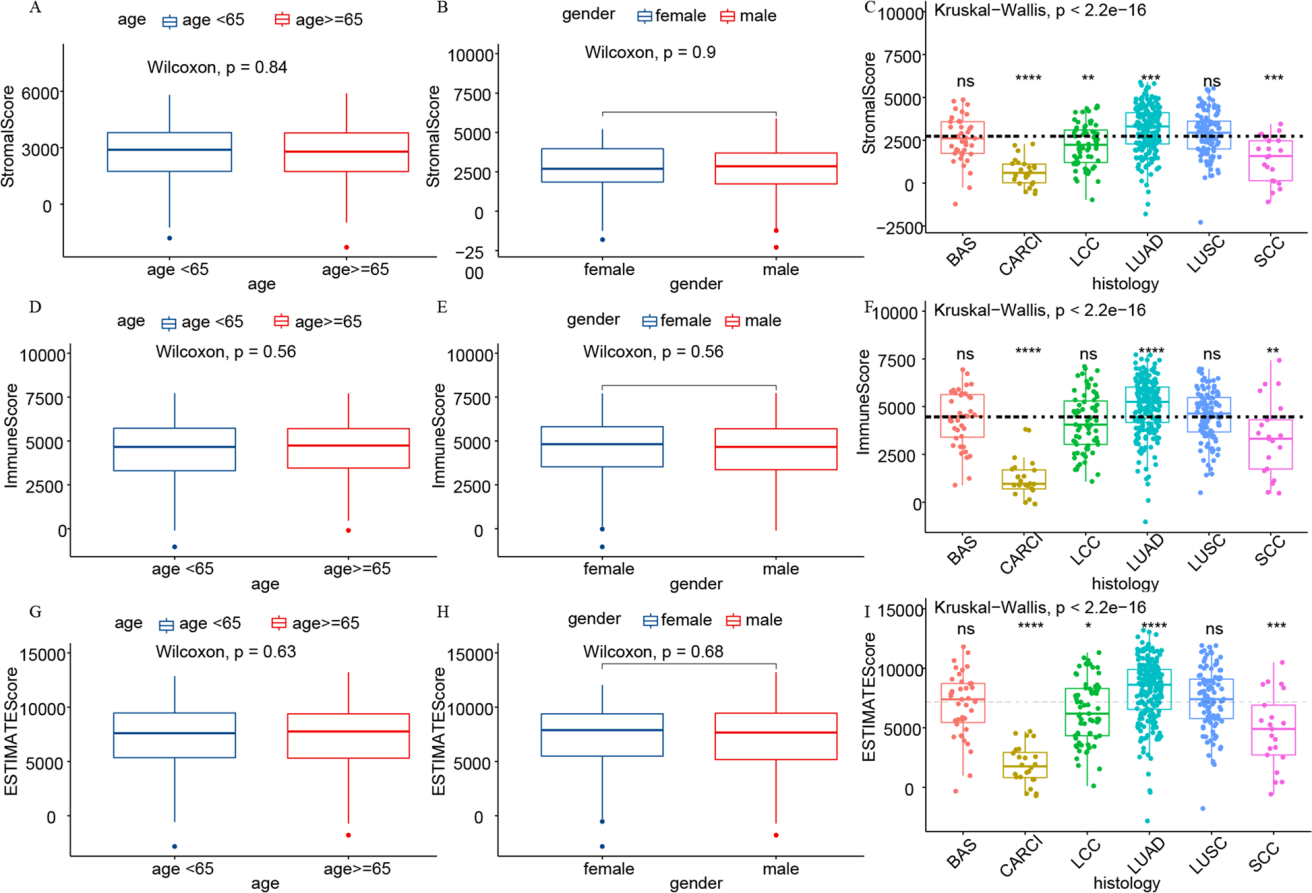
Correlation of Immune, Stromal, and ESTIMATE scores with base line characteristics. **A**-**C** Distribution of Immune score, Stromal score, and ESTIMATE score depending on age with *p* = 0.84, 0.56, and 0.63, respectively, using the Wilcoxon rank sum test. **D**-**F** Distribution of Immune score, Stromal score, and ESTIMATE score depending on gender with *p* = 0.90, 0.56, and 0.68, respectively, using the Wilcoxon rank sum test. **G**-**I** Distribution of Immune score, Stromal score, and ESTIMATE score depending on histology with *p < *2.2e^−16^, 2.2e^−16^, and 2.2e^−16^, respectively, using the Kruskal–Wallis rank sum test

**Fig. 3:**
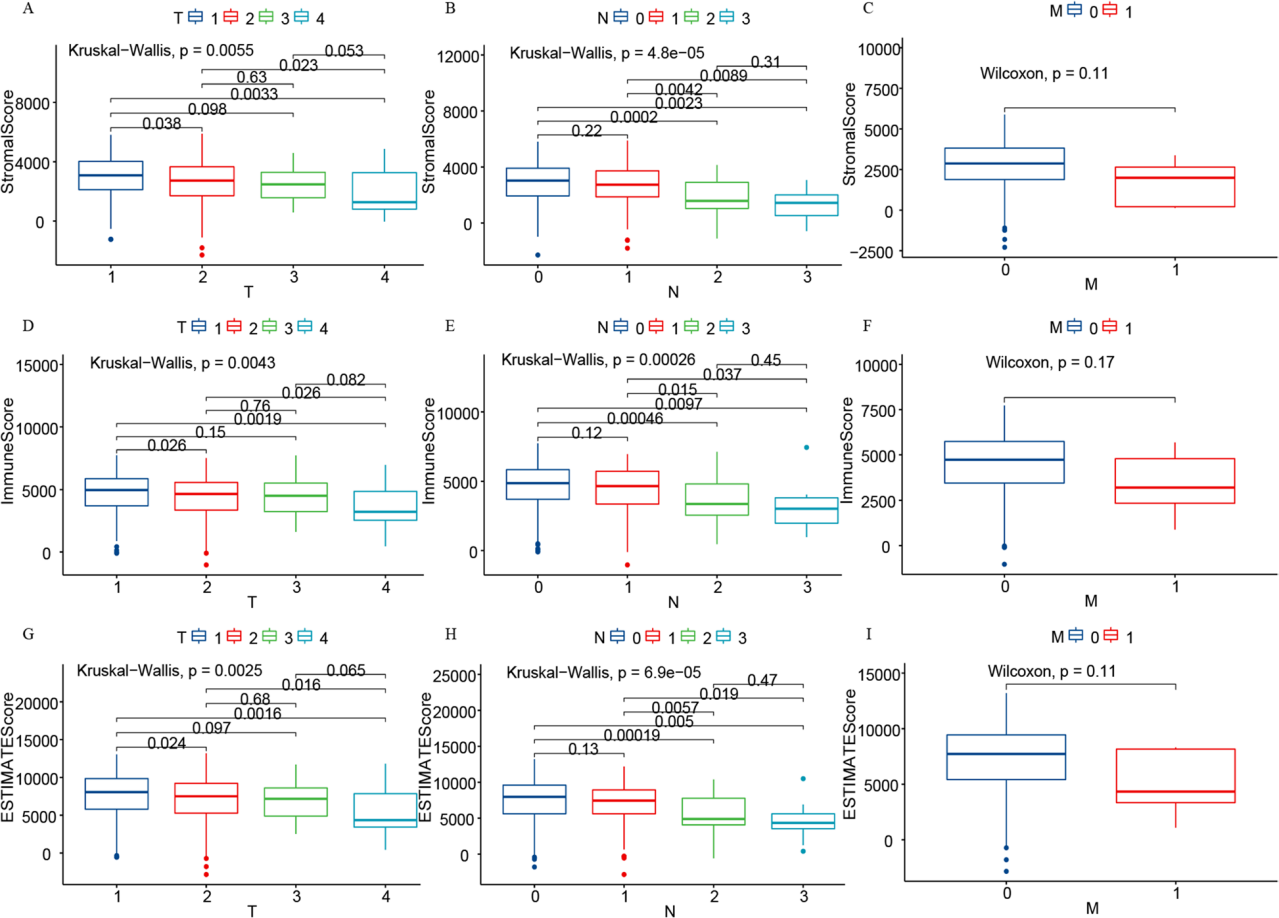
Correlation of Stromal, Immune, and ESTIMATE scores with clinical characteristics. **A**-**C** Distribution of Stromal score, Immune score, and ESTIMATE score depending on the T classification with *p* = 2.2e^−5^, 0.0043, and 0.0025, respectively, using the Kruskal–Wallis rank sum test. **D**-**F** Distribution of Stromal score, Immune score, and ESTIMATE score depending on the N classification with *p* = 0.0055, 0.00026, and 6.9e^−5^, respectively, using the Kruskal–Wallis rank sum test. **G**-**I** Distribution of Stromal score, Immune score, and ESTIMATE score depending on M classification with *p* = 0.11, 0.17, and 0.11, respectively, using the Wilcoxon rank sum test

We assessed the relationship between the patients' immune, stromal, and ESTIMATE scores and patient survival (both overall survival (OS) and disease-free survival (DFS)), using median pairs to classify these patients into high- and low-score groups. We found no difference (For OS, Immune score *p* = 0.72, Stromal score *p* = 0.52, ESTIMATE score *p* = 0.82; for DFS, Immune score *p* = 0.60, Stromal score *p* = 0.43, ESTIMATE score *p* = 0.92) in OS and DFS for lung cancer patients among all three scoring methods. However, we observed a trend towards better survival in patients with higher immune and ESTIMATE scores than in those with lower scores (Fig. 1). We then analyzed the clinicopathological characteristics and found no significant differences between the scores and the age, gender, as well as the AJCC-M stage of lung cancer patients. However, there were significant differences in the AJCC-T and -N stages of the patients as well as their pathological type, regardless of the scoring method (Figs. 2 and 3).

### Screening DEGs based on immune, stromal, and ESTIMATE scores

Differential expression analysis identified 114 DEGs based on their Immune, Stromal, and ESTIMATE scores (with |log FC|≥ 1.5 and FDR < 0.05 (Supplement Table 2)). Venn diagrams were constructed to show common up- and down-regulated DEGs that shared similar Immune, Stromal, and ESTIMATE scores (Supplement Fig. 2A). In the volcano plots of the DEGs, red dots represent significantly upregulated genes, green dots represent significantly downregulated genes, and black dots represent genes with no significant differences (Supplement Fig. 2B).

### Roles of DEGs during lung cancer pathogenesis

We analyzed the roles of DEGs during lung cancer pathogenesis by conducting GO enrichment analysis. The top 10 biological processes that involve these DEGs are shown in Supplement Fig. 2C–E. Among others, external side of plasma membrane, cytoplasmic vesicle lumen, and vesicle lumen were terms enriched in the cellular component (CC) category. The biological process (BP) category included humoral immune response, leukocyte migration, and adaptive immune response. For the molecular function (MF) category, DEGs were related to antigen binding, chemokine receptor binding, and serine − type endopeptidase activity. Through KEGG analysis, we determined that phagosome and cytokine − cytokine receptor interactions were associated with DEGs in lung cancer (Supplement Fig. 3A). A phagosome is a vesicle that forms around a particle engulfed by a phagocyte during phagocytosis. Professional phagocytes include macrophages, neutrophils, and dendritic cells (DCs). These cells are all involved in the body's immune response. We first observed the cytokine − cytokine receptor interaction, which was based on immune cells, and then analyzed the protein–protein interaction network (Supplement Fig. 3C). In order to explore whether the signaling pathways identified by GO and KEGG analyses were up-regulated or down-regulated, we performed gene set enrichment analysis (GSEA) using the entire matrix of DEGs. Our results showed that signaling pathways involved in hallmark allograft rejection, hallmark apical junction, hallmark interferon gamma response, the hallmark P53 pathway, and the hallmark TNF-α signaling via NF-ĸB were upregulated in high ESTIMATE score group (Supplement Fig. 3B).

### A prognostic model constructed based on immune-related genes

**Fig. 4:**
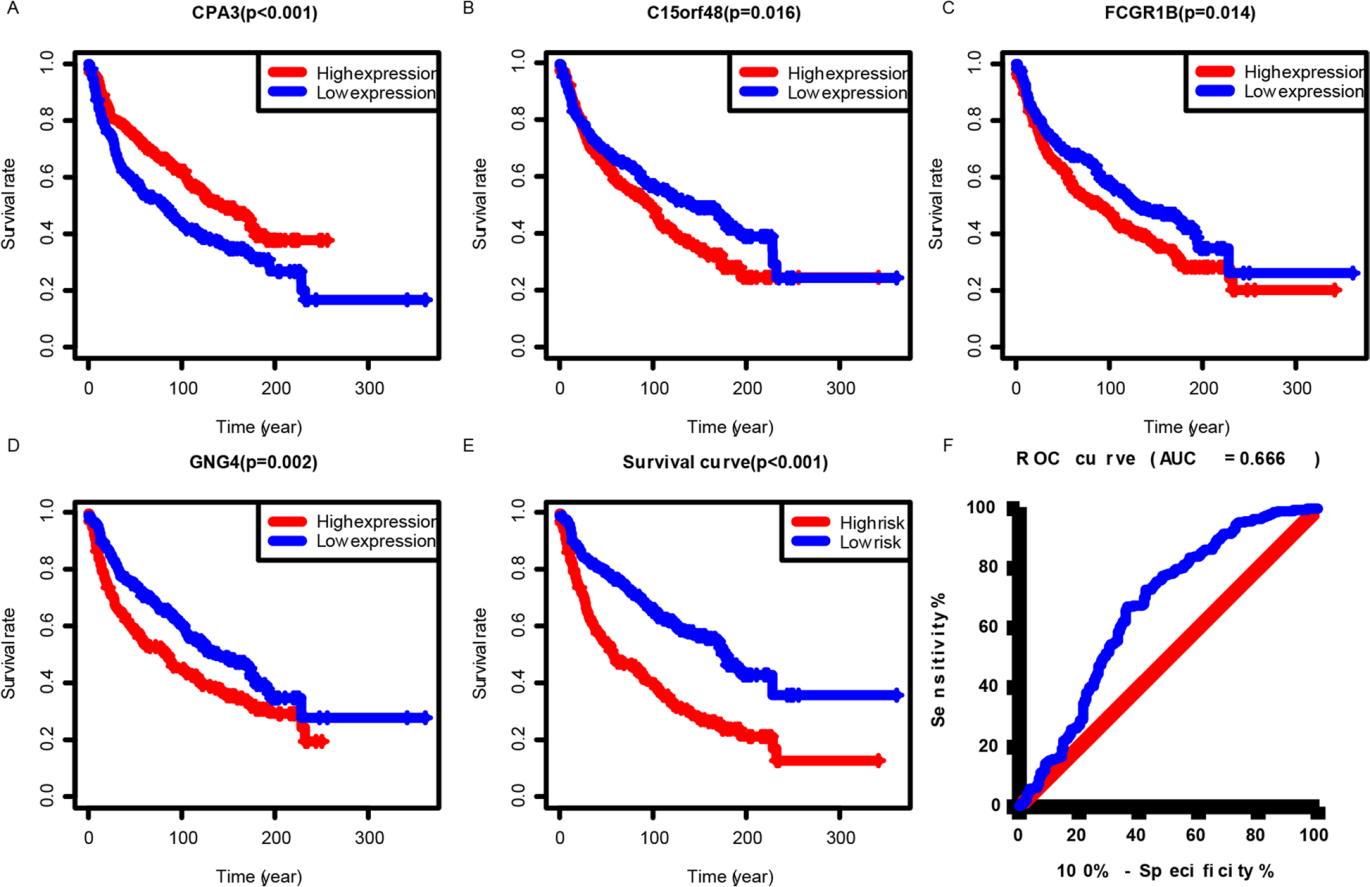
Overall survival curves obtained using the Kaplan–Meier method indicate that the risk score is significantly associated with OS prognosis. Kaplan–Meier survival analyses for lung cancer patients classified into groups with high or low expression of: **A ***CPA3* (*p* < 0.001 by log-rank test). **B ***C15orf48* (*p* = 0.016 by log-rank test). **C ***FCGR1B* (*p* = 0.014 by log-rank test). **D ***GNG4* (*p* = 0.002 by log-rank test). **E** Kaplan–Meier survival analysis for lung cancer patients classified into high- or low-risk groups for these four genes (*p* < 0.001 by log-rank test). **F** The horizontal and vertical axes represent false positive and true positive rates, respectively. The AUC value for the risk model in predicting the survival was 0.666. ROC, receiver operating characteristic; AUC, area under the curve

In the training set, we selected 12 genes that were in common between 20,174 prognostic immune genes and DEGs with |Log FC|≥ 1.5. First, we used univariate Cox proportional regression to select survival-related immune genes that included *ABI3BP*, *ADH1B*, *C15orf48*, *CPA3*, *CXCL9*, *FCGR1B*, *GNG4*, *NAPSA*, *PIGR*, *SFTA2*, *SFTPD*, and *SLC34A2* (*p* < 0.05). Of these 12 genes, a signature including the four genes *CPA3*, *C15orf48*, *FCGR1B*, and *GNG4* was identified by multivariate analysis (Fig. 4A–D). The risk score was calculated as (0.001) × (expression of *CPA3*) + (-0.016) × (expression of *C15orf48*) + (-0.014) × (expression of *FCGR1B*) + (-0.002) × (expression of *GNG4*). Then, the patient data were divided into two groups, patients with a high-risk score were found to have a poor survival (Fig. 4E). The area under the ROC curve (AUC) for the risk formula in the training set was 0.666 that confirmed predictivity of the signature (Fig. 4F). The C-index of the training set was 0.634 (95% CI: 0.616–0.652). These four genes may be able to predict the prognosis of lung cancer. Data from the TCGA database was used to validate the model. The data for lung cancer in the TCGA database do not include the full range of pathology types included in the model. Data for patients with NSCLC in the model represent 67.5% of the total. The remaining BAS, CARCI, LCC and SCC together, accounted for only 32.5%. Therefore, we only used the NSCLC data from TCGA for partial validation. The validation set C index was 0.631 (95% CI: 0.580–0.652). The area under the ROC curve (AUC) for the risk formula in the validation set was 0.560 that confirmed predictivity of the signature (Supplement Fig. 4).

### Profiles of TICs in tumor samples and correlation analyses

**Fig. 5:**
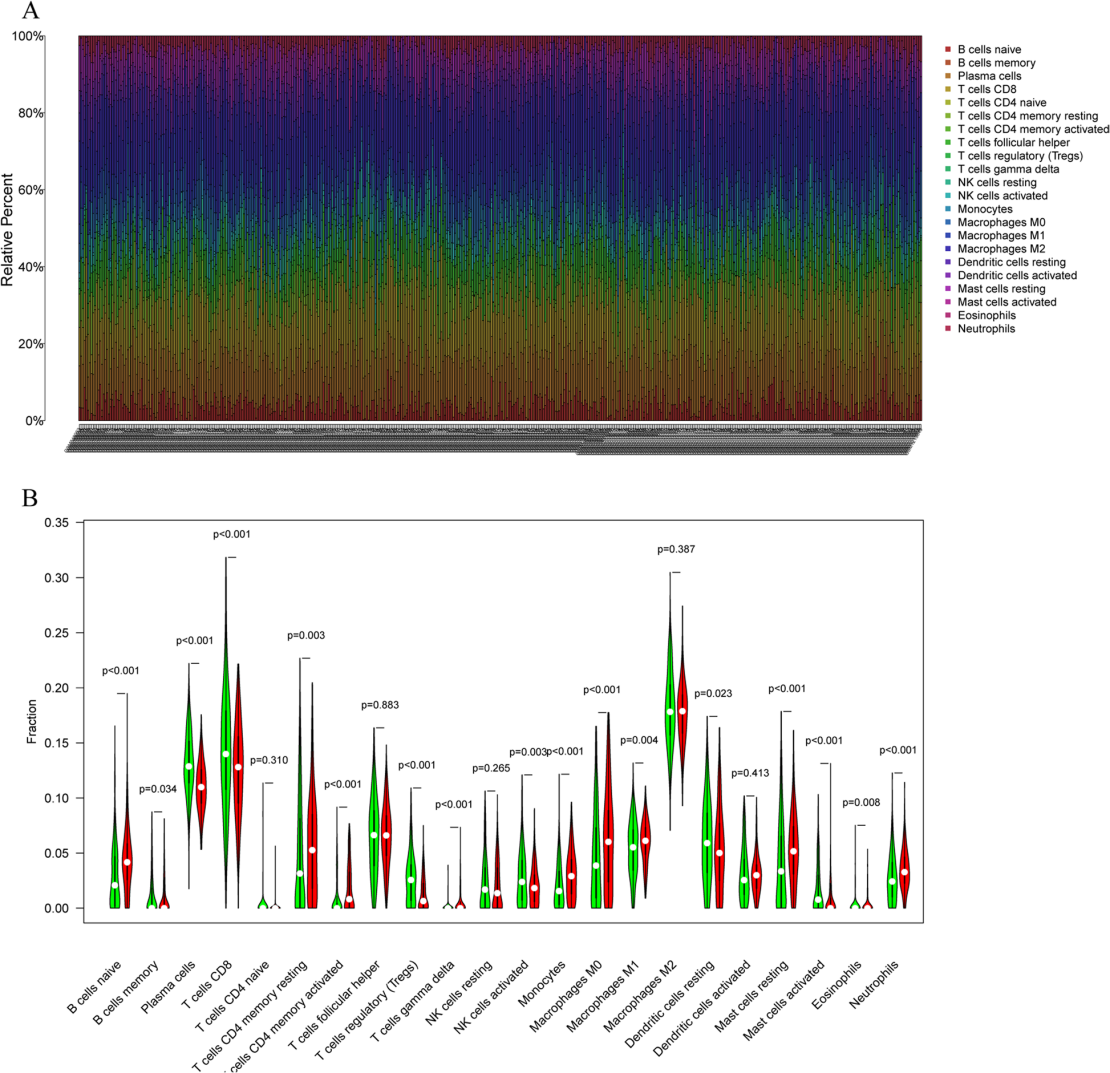
TIC profile in tumor samples and correlation analysis. **A** Bar plot showing the proportion of the 22 TIC types in lung cancer tumor samples. Column names in the plot represent the sample IDs. **B** Violin plot showing the ratio differentiation of the 22 immune cell types from lung tumor samples of patients with low or high ESTIMATE scores; the Wilcoxon rank sum was used to determine statistical significance

In order to confirm the relationship between tumor immunity and the tumor microenvironment in lung cancer patients, we used the CIBERSORT algorithm to assess the infiltration of 22 types of immune cells in each patient. We also analyzed the differences in immune cell infiltration between the two patient groups divided based on the ESTIMATE score. We found that memory B cells, plasma cells, CD8^+^ T cells, regulatory T cells, activated NK cells, resting DCs, activated mast cells, and eosinophil expression were elevated in the group with high ESTIMATE scores. Naïve B cells, memory B cells, CD4^+^ memory resting T cells, CD4^+^ memory activated T cells, monocytes, M1 macrophages, activated mast cells, and neutrophils were found to be elevated in the group with low ESTIMATE scores (Fig. 5). The ESTIMATE score corresponded to differences in the expression of immune cells in different tumor microenvironments.

### Different prognoses for different types of pathologies

**Fig. 6:**
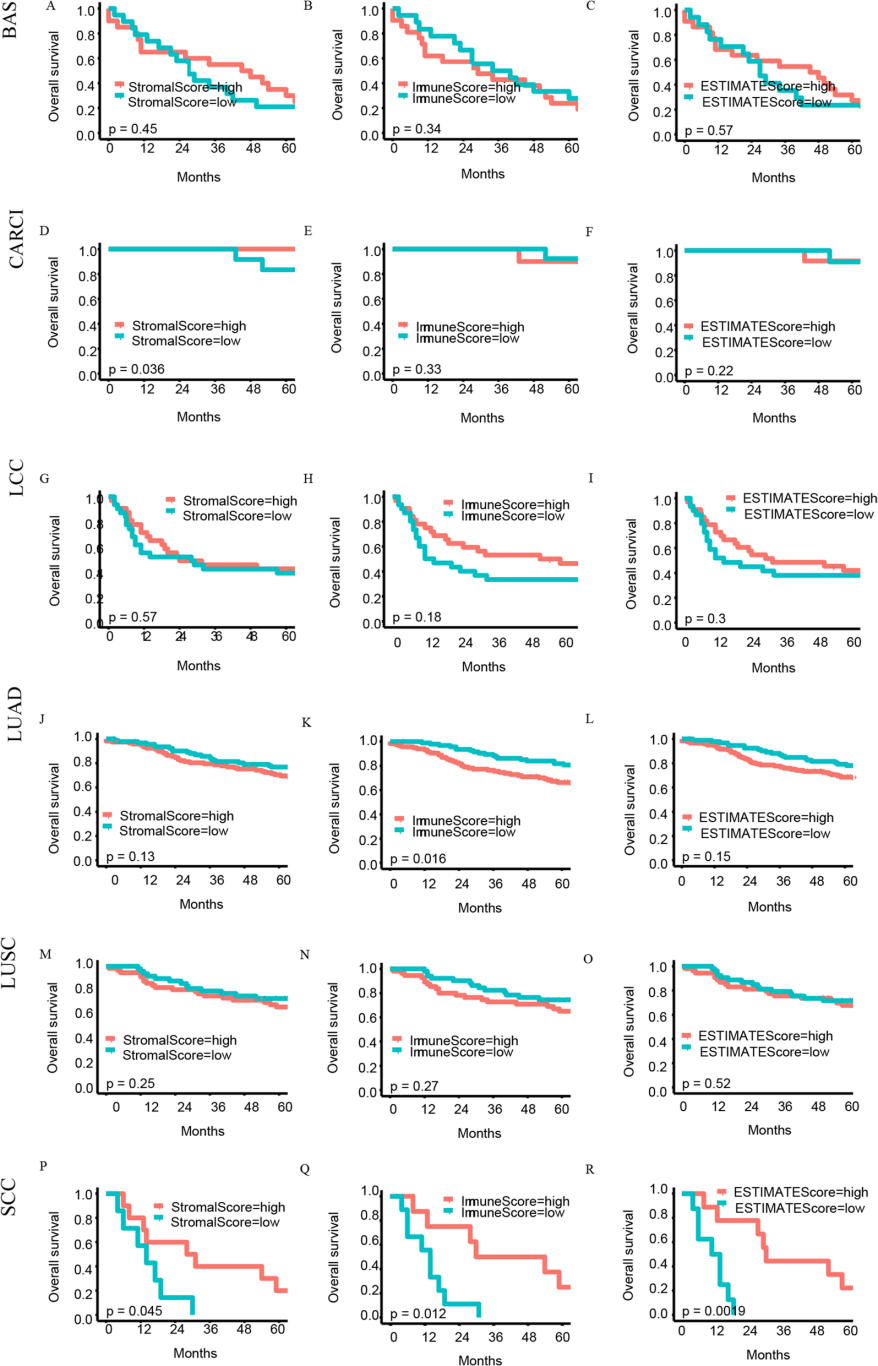
Correlation of the three scores with the survival of patients with different types of histology. BAS, basaloid; CARCI, carcinoid tumors; LCC, large cell cancer; LUAD, lung adenocarcinoma; LUSC, lung squamous cell carcinoma; SCC, small cell carcinoma

In this study, we observed a trend towards better survival in lung cancer patients with higher scores than in those with lower scores (Fig. 1). However, there were no statistically significant differences. Therefore, we analyzed associations between different pathological types of lung cancer and prognosis (Fig. 6). We discovered that the Stromal score predicted the prognosis of patients with CARCI; according to Kaplan–Meier analysis, patients in the high-Stromal-score group had better survival outcomes than those in the low-Stromal-score group (*p* = 0.036) (Fig. 6D). Moreover, the Immune score could predict the prognosis of LUAD patients; according to Kaplan–Meier analysis, patients in the low-Immune-score group had better survival outcomes than those in the high-Immune-score group (*p* = 0.016) (Fig. 6K). We also found that all the scoring methods predicted the outcome of SCC patients (Stromal score *p* = 0.045, Immune score *p* = 0.012, and ESTIMATE score *p* = 0.0019, Fig. 6P–R).

## Discussion

The high incidence and mortality rate of lung cancer have resulted in numerous studies on various aspects of lung cancer, including its pathogenesis, diagnosis, staging, treatment, and prognosis. Even after using a combination of surgery, radio- and chemotherapy, targeted therapy, and immunotherapy, the prognosis of lung cancer patients has remained poor. This might be related to the large number of pathological types of lung cancer, the late stage at detection, and the complexity of the tumor microenvironment. Existing indicators are still inadequate in predicting the prognosis of patient survival. Therefore, we explored the way in which immune and stromal scores were related to patient prognosis. We developed a prognostic model based on four genes associated with prognosis that could predict the prognosis of patients relatively well.

As shown in Fig. 1, we evaluated the relationship between the different scores and the survival prognosis of lung cancer patients. Although there were no statistically significant scores, the data we analyzed contained multiple lung cancer pathology types with different biological manifestations and prognoses that need to be considered. Further analysis revealed that Stromal score could predict the prognosis of CARCI patients (Fig. 6D), and Immune score could predict the prognosis of LUAD patients (Fig. 6K). All three scoring methods could predict the prognosis of SCC patients (Fig. 6P–R). Interestingly, in contrast to our findings, these three scoring modalities have been shown to be predictive of prognosis in lung cancer patients [22–24]. These differences could be potentially caused by a low number of cases in each subgroup in our current study as well as our more specific classification of the different pathological types, which may reflect the state of the different tumor microenvironments. Analysis of the relationship between clinical characteristics and these scores revealed that Stromal, Immune, and ESTIMATE scores varied considerably across pathological types, which supported the results of our survival analysis of different lung cancer pathology types. These scores did not seem to accurately predict the prognosis of lung cancer patients; therefore, we examined genes representing different scores and identified 114 DEGs in total. Cox regression analysis allowed us to identify four genes that were associated with lung cancer prognosis. A model consisting of these four genes was able to predict the prognosis of lung cancer patients relatively well with an AUC of 0.666. Results of the GSEA as well as the GO and KEGG analyses based on lung cancer RNA-seq data supported the relevance of these DEGs to lung cancer immunity.

Based on the classification of the Trading Card Database (http://www.tcdb.org/), monovalent cation proton antiporter (CPA) superfamily is now divided into three CPA families: CPA1, CPA2, and CPA3 [25]. According to this current classification, the CPA2 and CPA3 families include primarily bacterial, fungal, and plant transporter proteins [26]. Visser et al. concluded that in patients who had early or locally advanced NSCLC (96.3%). Gene expression profiling revealed five markers, which mRNA levels strongly correlated to pemetrexed target genes mRNA levels: TPX2, CPA3, EZH2, MCM2 and TOP2A [27]. The expression of CPA3 in NSCLC patients differs from their response to chemotherapy, and the difference in treatment efficacy will directly result in a poorer prognosis than in patients who responded. Mirjana et al. found that the combined action of mast cell chymase, tryptase and CPA3 protects against melanoma colonization of the lung. This indicated that CPA3 played a key role in lung tumors, and the combination of CPA3 with other genes could accurately predict the prognosis of patients with lung cancer [28]. Previous studies, all of which highlighted CPA3's importance in lung cancer, supported our conclusion. The *C15orf48* gene was first identified while studying human esophageal squamous cell carcinoma tissue. Both mRNA and protein levels of *C15orf48* were reduced in the cancer cell samples [29, 30]. Lee et al. uncovered a dual-component pleiotropic regulation of host inflammation and immunity by *C15orf48* to safeguard the host during infection and inflammation [31]. The *C15orf48* gene may be significantly involved in lung cancer immunotherapy, which may influence the outcome in lung cancer patients. *FCGR1B* (Fc Fragment of IgG Receptor Ib) is a pseudogene that binds the Fc region of IgGs with a low affinity than *FCGR1A*, and may have a function in the humoral immune response [32, 33]. *GNG4* encodes receptors on cell membranes that sense various signals from neurotransmitters, hormones, and light. Most of these receptors are coupled to G proteins that transduce the signal to effectors [34]. GNG4 expression was elevated in LUAD, and GNG4 overexpression was associated with poor prognosis in LUAD patients. The hypoxic microenvironment of lung adenocarcinoma could promote the expression of GNG4, and GNG4 promoted the migration and proliferation of LUAD cells [35]. In our article, we found that these genes are associated with the prognosis of lung cancer patients, which to some extent enriches the study of these genes and gives us a more comprehensive understanding of their functions.

We analyzed the infiltrating immune-cell profiles in patients and ultimately found that differences in the ESTIMATE score represented different levels of infiltration of various immune cells. In this study, various immune cells had differential expression profiles, thereby elucidating that different tumor microenvironments had different cell types of varying proportions. These differences are likely to play a crucial role in eventual remission in patients. There is still a lot to explore about the immune microenvironment of tumors. We believe that in the future we will continue to research not only the composition and function of immune cells, but also their interactions with tumor cells.

Our study investigated the immune infiltration status, cell composition, and prognosis of lung cancer patients with different scores by analyzing differential gene expression and related pathways using RNA-seq data. Using the background from previous studies, we screened DEGs and developed a model based on 4 genes to predict patient prognosis. We have validated the model using data from the TCGA database and demonstrated that our model had some predictive power. Patients with various pathological types were evaluated based on the three scoring methods. Although our study used data from an online database and reduced the socio-economic burden of sequencing, it still has some shortcomings. First, our study was limited by the type of data entered into the database, and even though best efforts were made to eliminate batch effects, there was no way for us to completely remove batch effects inherent in each dataset. Second, although we included the data of 474 patients in our analyses, the low number of cases in each subgroup may have skewed some results. Thirdly, in addition to lacking an external validation set, we neither validated the basal levels of screened genes nor performed further flow cytometry analyses on the immune cells that were screened for differential expression. Due to the lack of data on lung cancer including all pathological types in the TCGA database, we only partially validated the model for 67.5% of NSCLC that occupied the model. Those were the problems we discussed in our article, and we hoped to use them to inspire future research. Finally, although we partially validated the validity of the model, the absence of patient treatment data in our included data was a limiting factor of our study.

A dataset obtained from a public database was used to evaluate the ESTIMATE score. And we conducted differential expression analysis and made a risk formula with four DEGs, which could distinguish patients with better or worse prognosis.

In conclusion, we found that Immune, Stromal, and ESTIMATE scores could predict the prognosis in a proportion of lung cancer patients. We constructed a model to assess the prognosis of lung cancer patients based on DEGs and assessed the degree of immune cell infiltration in lung cancer patients with different scores.

## Supplementary Information

**Additional file 1: Supplement Fig. 1.** Overall flowchart of steps involved in construction of the prognostic metabolic gene signature.

**Additional file 2: Supplement Fig. 2.** Venn plot, hot plot, and GO enrichment analysis for the DEGs.

**Additional file 3: Supplement Fig. 3.** KEGG, GSEA, and PPI network analyses.

**Additional file 4: Supplement Fig. 4.** ROC curve of the validation set.

**Additional file 5: Supplement Table 1.** Basic characteristics of the patients.

**Additional file 6: Supplement Table 2.** The list of differential expression analysis identified 114 DEGs based on their Immune, Stromal, and ESTIMATE scores.

## Data Availability

Publicly available datasets were analyzed in this study. The datasets analyzed are available in the below: https://www.ncbi.nlm.nih.gov/geo/query/acc.cgi?acc=GSE30219, https://www.ncbi.nlm.nih.gov/geo/query/acc.cgi?acc=GSE50081. All data used during this study are available from the corresponding authors upon reasonable request.

## Abbreviations

TICs: Tumor-infiltrating immune cells
WHO: The World Health Organization
ICIs: Programmed death-1
PD-1: Immune checkpoint inhibitors
PD-L1: Programmed death-ligand 1
AUC: The Area Under Curve
GEO: The Gene Expression Omnibus
BAS: Basaloid
CARCI: Carcinoids
LCC: Large cell tumor
LUAD: Lung adenocarcinoma
LUSC: Lung squamous cell carcinoma
SCC: Small cell carcinoma tissue
DEGs: Differentially expressed genes
GO: Gene ontology
KEGG: KOBAS-Kyoto Encyclopedia of Genes and Genomes
CC: Cellular component
BP: Biological process
MF: Molecular function
