# Supplementary material for: Four differentially expressed genes can predict prognosis and microenvironment immune infiltration in lung cancer: a study based on data from the GEO
Source: BMC Cancer. 2022 Feb 21;22:193. doi: 10.1186/s12885-022-09296-8 (PMC8859904; doi:10.1186/s12885-022-09296-8)
Supplement: Supplementary file 1 — Additional file 1: Supplement Fig. 1. Overall flowchart of steps involved in construction of the prognostic metabolic gene signature. [file 12885_2022_9296_MOESM1_ESM.pdf]

Supplement Figure 1. Overall flowchart of steps involved in construction of the prognostic metabolic gene signature.

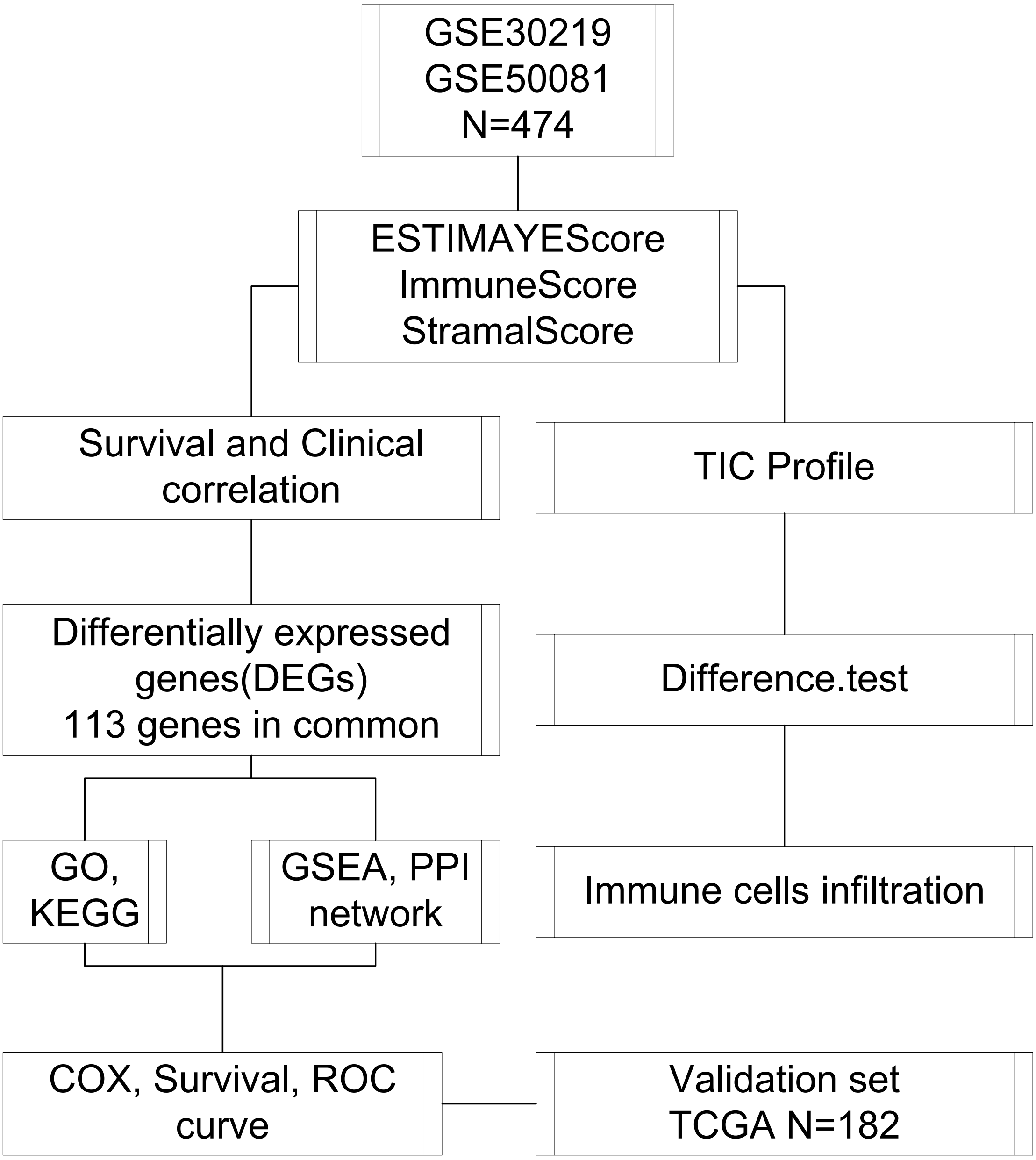

Supplement Figure 1. TIC = Tumor-infiltrating Immune Cells; GO = Gene Ontology Analysis; KEGG = Kyoto Encyclopedia of Genes and Genomes; GSEA = Gene Set Enrichment Analysis; PPI network = Protein-Protein Interaction Network. COX = Cox Regression Analysis; ROC curve = Receiver Operating Characteristic.
