# Supplementary material for: Four differentially expressed genes can predict prognosis and microenvironment immune infiltration in lung cancer: a study based on data from the GEO
Source: BMC Cancer. 2022 Feb 21;22:193. doi: 10.1186/s12885-022-09296-8 (PMC8859904; doi:10.1186/s12885-022-09296-8)
Supplement: Supplementary file 2 — Additional file 2: Supplement Fig. 2. Venn plot, hot plot, and GO enrichment analysis for the DEGs. [file 12885_2022_9296_MOESM2_ESM.pdf]

Supplement Figure 2. Venn plot, hot plot, and GO enrichment analysis for the DEGs.

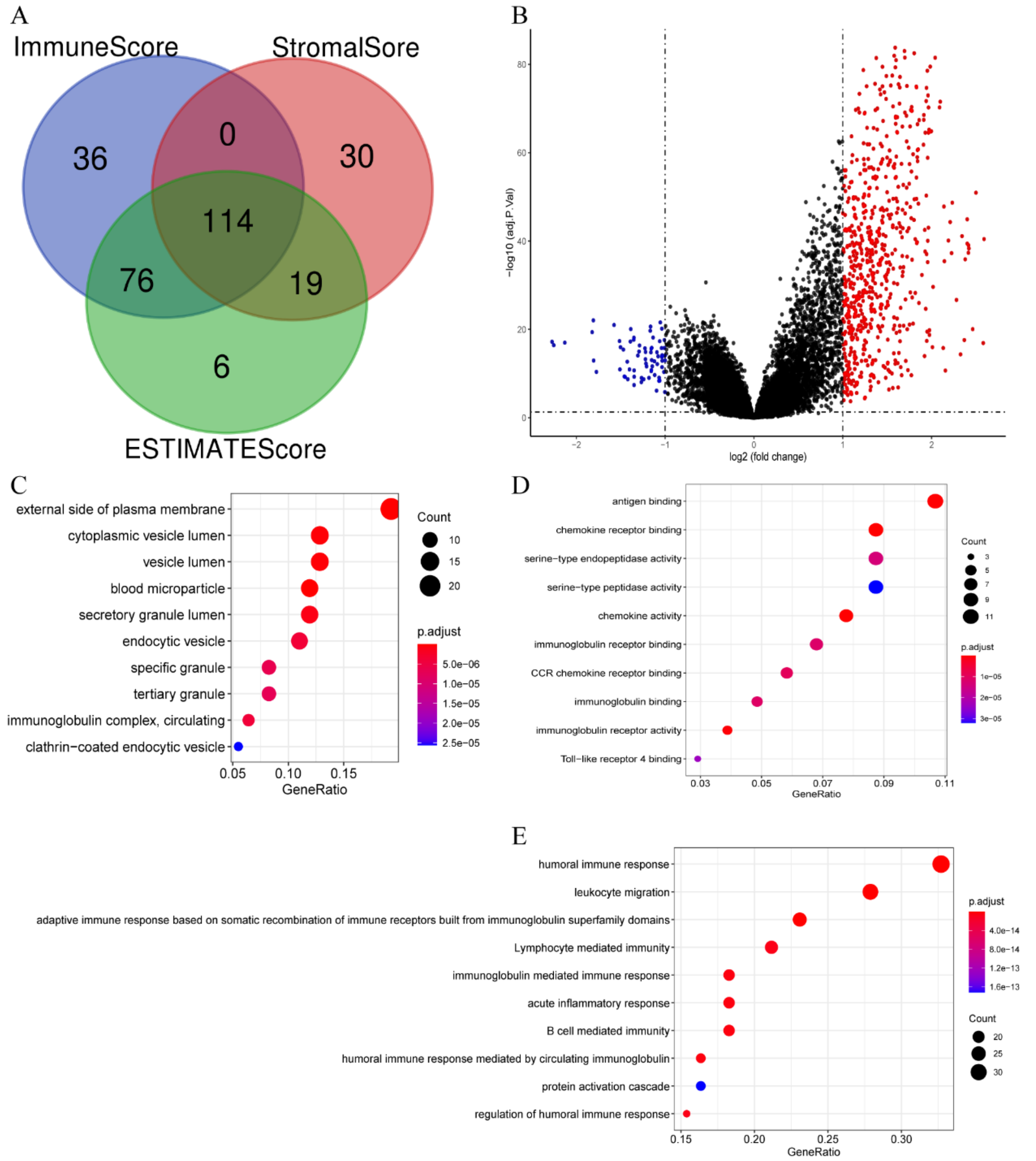

Supplement Figure 2. A. Venn plots showing common up- and down-regulated DEGs that share Immune, Stromal and ESTIMATE scores; $q < 0.05$ and $|\log \text{fold-change}| > 1.5$ were the filtering thresholds for significance of DEGs. B. Hot plot for DEGs. C—E. GO enrichment analysis for 114 DEGs, terms with p and $q < 0.05$ were considered to be enriched significantly. C. Cellular component (CC). D. Molecular function (MF). E. Biological process (BP).
