# Supplementary material for: Four differentially expressed genes can predict prognosis and microenvironment immune infiltration in lung cancer: a study based on data from the GEO
Source: BMC Cancer. 2022 Feb 21;22:193. doi: 10.1186/s12885-022-09296-8 (PMC8859904; doi:10.1186/s12885-022-09296-8)
Supplement: Supplementary file 3 — Additional file 3: Supplement Fig. 3. KEGG, GSEA, and PPI network analyses. [file 12885_2022_9296_MOESM3_ESM.pdf]

Supplement Figure 3. KEGG, GSEA, and PPI network analyses.

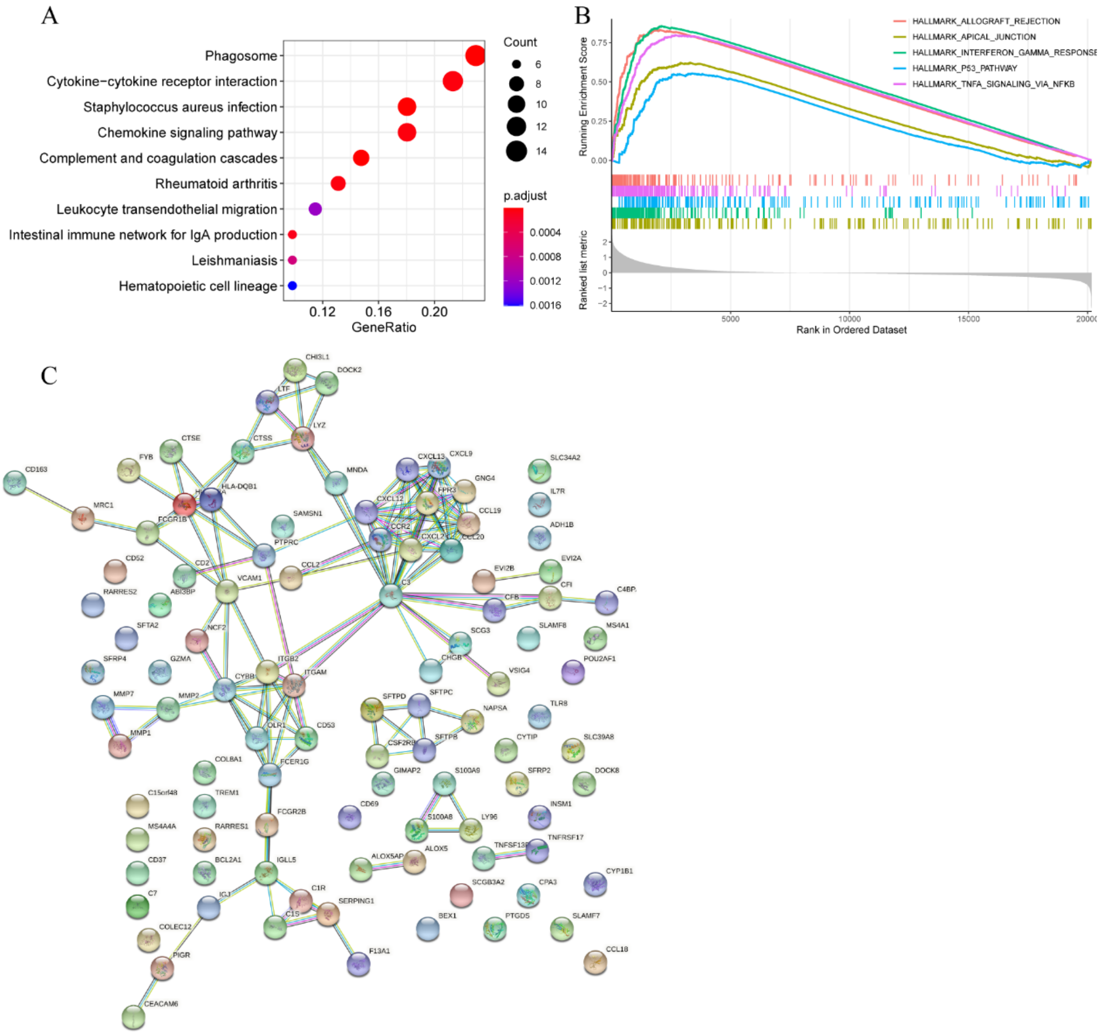

Supplement Figure 3. KEGG, GSEA, and PPI network analyses. A. KEGG pathway analysis. The color and size of the circles were determined by the degree value with red circles representing upward, and blue circles representing downward. B. GSEA for samples with high ESTIMATE scores.C. PPI network analysis for 114 DEGs.
