# Supplementary material for: Four differentially expressed genes can predict prognosis and microenvironment immune infiltration in lung cancer: a study based on data from the GEO
Source: BMC Cancer. 2022 Feb 21;22:193. doi: 10.1186/s12885-022-09296-8 (PMC8859904; doi:10.1186/s12885-022-09296-8)
Supplement: Supplementary file 4 — Additional file 4: Supplement Fig. 4. ROC curve of the validation set. [file 12885_2022_9296_MOESM4_ESM.pdf]

Supplement Figure 4. ROC curve of the validation set.

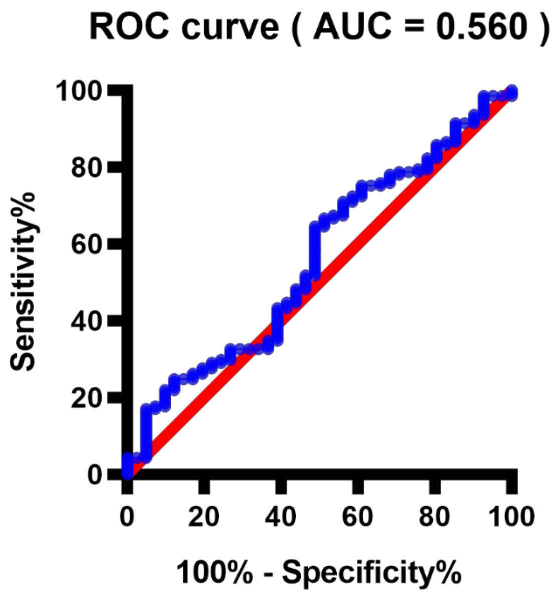

Supplement Figure 4. ROC curve of the validation set. The horizontal and vertical axes represent false positive and true positive rates, respectively. The AUC value for the validation set was 0.560. ROC, receiver operating characteristic; AUC, area under the curve.
