# Supplementary material for: Four differentially expressed genes can predict prognosis and microenvironment immune infiltration in lung cancer: a study based on data from the GEO
Source: BMC Cancer. 2022 Feb 21;22:193. doi: 10.1186/s12885-022-09296-8 (PMC8859904; doi:10.1186/s12885-022-09296-8)
Supplement: Supplementary file 5 — Additional file 5: Supplement Table 1. Basic characteristics of the patients. [file 12885_2022_9296_MOESM5_ESM.pdf]

Supplement Table 1. Basic characteristics of the patients.

	ImmuneScore		StromalScore		ESTIMATEScore	
	**high (N=346)**	**low (N=128)**	**high (N=320)**	**low (N=154)**	**high (N=328)**	**low (N=146)**
**Gender**						
female	94 (27.2%)	32 (25.0%)	86 (26.9%)	40 (26.0%)	89 (27.1%)	37 (25.3%)
male	77 (22.3%)	21 (16.4%)	70 (21.9%)	28 (18.2%)	74 (22.6%)	24 (16.4%)
man	175 (50.6%)	75 (58.6%)	164 (51.3%)	86 (55.8%)	165 (50.3%)	85 (58.2%)
**Age**						
age <55	58 (16.8%)	30 (23.4%)	56 (17.5%)	32 (20.8%)	56 (17.1%)	32 (21.9%)
age>=55	288 (83.2%)	97 (75.8%)	264 (82.5%)	121 (78.6%)	272 (82.9%)	113 (77.4%)
Missing	0 (0%)	1 (0.8%)	0 (0%)	1 (0.6%)	0 (0%)	1 (0.7%)
**histology**						
BAS	28 (8.1%)	11 (8.6%)	25 (7.8%)	14 (9.1%)	25 (7.6%)	14 (9.6%)
CARCI	2 (0.6%)	22 (17.2%)	2 (0.6%)	22 (14.3%)	0 (0%)	24 (16.4%)
LCC	41 (11.8%)	26 (20.3%)	35 (10.9%)	32 (20.8%)	36 (11.0%)	31 (21.2%)
LUAD	181 (52.3%)	32 (25.0%)	170 (53.1%)	43 (27.9%)	177 (54.0%)	36 (24.7%)
LUSC	83 (24.0%)	23 (18.0%)	77 (24.1%)	29 (18.8%)	79 (24.1%)	27 (18.5%)
SCC	8 (2.3%)	13 (10.2%)	7 (2.2%)	14 (9.1%)	7 (2.1%)	14 (9.6%)
Other	3 (0.9%)	1 (0.8%)	4 (1.3%)	0 (0%)	4 (1.2%)	0 (0%)
**T**						
1	174 (50.3%)	49 (38.3%)	170 (53.1%)	53 (34.4%)	170 (51.8%)	53 (36.3%)
2	140 (40.5%)	51 (39.8%)	120 (37.5%)	71 (46.1%)	128 (39.0%)	63 (43.2%)
3	22 (6.4%)	11 (8.6%)	21 (6.6%)	12 (7.8%)	21 (6.4%)	12 (8.2%)
4	9 (2.6%)	12 (9.4%)	8 (2.5%)	13 (8.4%)	8 (2.4%)	13 (8.9%)
X	1 (0.3%)	5 (3.9%)	1 (0.3%)	5 (3.2%)	1 (0.3%)	5 (3.4%)

**N**						
0	254 (73.4%)	73 (57.0%)	239 (74.7%)	88 (57.1%)	241 (73.5%)	86 (58.9%)
1	76 (22.0%)	29 (22.7%)	68 (21.3%)	37 (24.0%)	74 (22.6%)	31 (21.2%)
2	12 (3.5%)	18 (14.1%)	11 (3.4%)	19 (12.3%)	11 (3.4%)	19 (13.0%)
3	4 (1.2%)	6 (4.7%)	2 (0.6%)	8 (5.2%)	2 (0.6%)	8 (5.5%)
X	0 (0%)	2 (1.6%)	0 (0%)	2 (1.3%)	0 (0%)	2 (1.4%)
**M**						
0	342 (98.8%)	121 (94.5%)	317 (99.1%)	146 (94.8%)	324 (98.8%)	139 (95.2%)
1	3 (0.9%)	5 (3.9%)	3 (0.9%)	5 (3.2%)	3 (0.9%)	5 (3.4%)
X	1 (0.3%)	2 (1.6%)	0 (0%)	3 (1.9%)	1 (0.3%)	2 (1.4%)

Supplement Table 1. BAS, basaloid; CARCI, carcinoid tumors; LCC, large cell cancer; LUAD, lung adenocarcinoma; LUSC, lung squamous cell carcinoma; SCC, small cell carcinoma. AJCC-T, -N and -M stages of the patients.
