# Supplementary material for: Four differentially expressed genes can predict prognosis and microenvironment immune infiltration in lung cancer: a study based on data from the GEO
Source: BMC Cancer. 2022 Feb 21;22:193. doi: 10.1186/s12885-022-09296-8 (PMC8859904; doi:10.1186/s12885-022-09296-8)
Supplement: Supplementary file 6 — Additional file 6: Supplement Table 2. The list of differential expression analysis identified 114 DEGs based on their Immune, Stromal, and ESTIMATE scores. [file 12885_2022_9296_MOESM6_ESM.pdf]

Supplement Table 2. Differential expression analysis identified 114 DEGs based on their Immune, Stromal, and ESTIMATE scores

Names	total	elements	
**ESTIMATEScore, ImmuneScore and StromalSore**	114	TAGAP	IGK
		IL7R	EVI2A
		SCG3	ABI3BP
		MMP2	DOCK8
		ITGAM	FCER1G
		SFRP2	C3
		CHGB	TRBC1
		SLC39A8	SFRP4
		CD163	VSIG4
		IGHD	BMS1P20
		GIMAP2	POU2AF1
		CD37	C4BPA
		VCAM1	NAPSA
		SFTPC	TNFSF13B
		C1S	SERPING1
		LINC01279	TREM1
		C1R	SAMSN1
		IGHM	CD69
		MMP7	CXCL12
		CTSS	ITGB2
		C15orf48	CTSE
		MS4A1	NCF2
		CCR2	HLA-DRA
		CEACAM6	EVI2B
		DOCK2	IGLC1
		ADH1B	CD52
		CCL18	CPA3
		IGLJ3	CXCL9
		FPR3	PIGR
		SFTA2	C7
		F13A1	S100A8
		CXCL13	CYTIP
		ALOX5	CYBB
		TLR8	LTF
		LYZ	MRC1
		RARRES1	SCGB3A2
		PTGDS	IGHG1
		INSM1	CHI3L1
		CFB	CD53
		FCGR2B	LY96

		TNFRSF17	SFTPB
		SLC34A2	CCL2
		CCL20	BEX1
		SLAMF8	MMP1
		RARRES2	COL8A1
		SFTPD	FCGR1B
		FYB1	HLA-DQB1
		ALOX5AP	COLEC12
		CFI	JCHAIN
		GZMA	CYP1B1
		OLR1	CSF2RB
		IGKC	MNDA
		MS4A4A	BCL2A1
		CD2	IGLV1-44
		CCL19	SLAMF7
		CXCL2	S100A9
		GNG4	PTPRC
**ESTIMATEScore and ImmuneScore**	76	CXCL10	LAMP3
		SPINK1	CCL8
		PDZK1IP1	C1QA
		LCP2	GBP1
		IL10RA	ITK
		CCL5	HLA-DPB1
		TYROBP	SCGB3A1
		HLA-DMA	SASH3
		SLA	CD74
		C16orf54	DNER
		PCSK1	C16orf89
		IL32	ICAM1
		TRAF3IP3	SNX20
		PLAAT4	APBB1IP
		TRIM22	GZMB
		CD84	LRRK2
		RNASE6	CHIT1
		CCR1	CD86
		HLA-DOA	GPR171
		HLA-DMB	AQP4
		GPNMB	CP
		LAPTM5	IGLL3P
		NAPSB	CHGA
		CD27	CXCL11
		ARHGAP30	C1QB

		KYNU	APOC1
		MPEG1	TRAC
		RAC2	C1orf162
		INA	ITGAL
		ADAMDEC1	GZMK
		SELL	CD48
		PIK3CG	CCR7
		SFTA3	CXCL17
		CALHM6	ST18
		IGSF6	FDCSP
		SYT13	HCLS1
		MZB1	MS4A6A
		CLEC7A	IL2RG
**ESTIMATEScore and StromalSore**	19	CLMP	CCDC80
		PLAU	CEMIP
		MOXD1	CDH11
		FCGR2A	GREM1
		CTSK	COMP
		COL10A1	SLC6A14
		LRRC15	NNMT
		DCN	PTGIS
		MXRA5	EFEMP1
		COL11A1	
**ImmuneScore**	36	CSTA	HLA-DOB
		GABRB3	CCL4
		CCL21	LST1
		SLC7A7	CTSH
		KLHL6	CLIC2
		IDO1	CD3D
		C1QC	CORO1A
		HLA-DPA1	P2RX7
		ANKRD22	TACSTD2
		LTB	HAVCR2
		ARHGAP9	THEMIS2
		LCP1	LPXN
		ACP5	SYT4
		CD8A	HLA-DQA1
		SCG2	GUSBP11
		NEFL	CARD16
		SLC35D3	PTGER4
		CD79A	RGS1
**StromalSore**	30	GAS1	VGLL3
		ADAM12	PTGS2

		DPT	CCN4
		LUM	ITGBL1
		LOX	COL5A1
		SULF1	FNDC1
		TNC	COL8A2
		SPON1	GJB2
		COL5A2	FBN1
		FAP	THBS2
		CXCL5	BICC1
		SERPINF1	GJA5
		INHBA	SEMA3C
		PDGFRA	COL12A1
		POSTN	PRRX1
**ESTIMATEScore**	6	STEAP4	FGG
		TGM2	TPSB2
		SDR16C5	NDNF

Supplement Table 2. The list of differential expression analysis identified 114 DEGs based on their Immune, Stromal, and ESTIMATE scores (with $|\log FC| \geq 1.5$ and $FDR < 0.05$).
